# Feto-Maternal Outcomes and Treatment Compliance in Metformin Versus Insulin-Treated Gestational Diabetic and Non-Diabetic Patients at the Rehman Medical Institute, Peshawar

**DOI:** 10.7759/cureus.17424

**Published:** 2021-08-25

**Authors:** Nouman Anthony, Athar Ahmad, Chaand Bibi, Wareesha Amirzadah, Spogmay Humayun, Mehwish Sajid, Zainab Ashraf, Maimoona Abid, Muhammad Hasnain Khan, Zaland A Yousafzai

**Affiliations:** 1 General Medicine, Rehman Medical Institue, Peshawar, PAK; 2 Internal Medicine, Rehman Medical Institute, Peshawar, PAK; 3 General Medicine, Rehman Medical Institute, Peshawar, PAK; 4 Obstetrics and Gynaecology, Lady Reading Hospital MTI, Peshawar, PAK

**Keywords:** fetal maternal outcomes, gestational diabetes mellitus, metformin, insulin, premature rupture of membranes, pregnancy induced hypertension, eclampsia

## Abstract

Introduction

Gestational diabetes mellitus (GDM) is defined as any degree of glucose intolerance with onset or first recognition during pregnancy. The diagnosis is made on the basis of the oral glucose tolerance test (OGTT) which according to the guidelines of ACOG regards a blood glucose level higher than 190mg/dL after the one-hour test as the criteria for GDM. The first-line agent for GDM is insulin injections; however, it has high costs and also causes its own feto-maternal complications which can include weight gain and polyhydramnios. On the contrary, metformin has fewer complications, is cheaper, and is emerging as a better alternative for the first-line agent for the treatment of diabetes mellitus type 2. GDM had a prevalence of 11.8% in the year 2018 in all trimesters of pregnancy in Pakistan. This study was thus conducted to determine the feto-maternal outcomes of non-GDM and GDM patients on insulin, metformin, and combined treatment respectively admitted to gynecology ward Rehman Medical Institute (RMI) Khyber Pakhtunkhwa, Pakistan in the year 2019.

Objectives

To determine the feto-maternal outcomes in patients of GDM on metformin treatment and the feto-maternal outcomes in patients of GDM on insulin treatment and to compare the feto-maternal outcomes of mothers with GDM to those without GDM.

Methodology

This is a retrospective study conducted from January to April 2020 on patients of gestational diabetes mellitus undergoing either metformin, insulin, or both therapies admitted to the gynecology ward, Rehman Medical Institute (RMI). After getting ethical approval from the institutional ethical approval board, data were collected for the entire year of 2019 on the basis of proforma with the variables: demographic data, glycemic control (via OGTT), mode of labor, primary open-angle glaucoma (POAG), and feto-maternal outcomes. Data was entered and analyzed via SPSS version 21.0 (IBM SPSS Statistics for Windows, IBM Corp., Armonk, NY) and the data were run through various tests including descriptive statistics, cross-tabulations, and chi-square. Results were formulated on the basis of these reports which were then presented in the form of graphs and tables.

Results

Out of 150 mothers who were admitted for delivery at the gynecology ward, 123 (82.0%) women were 30-40 years of age. Non-gestational diabetics patients were 78 (52%) whereas gestational diabetic mothers were 72 (48%); within these GDM-positive mothers 44 (61.1%) were on metformin, 21 (29.1%) were on insulin and seven (9.7%) were on combined treatment. Among modes of delivery, C-section was the most common (113 [76%]), mostly in non-GDM mothers (95 [45.1%]) followed by those on metformin treatment (36 [31.8%]). Considering fetal outcomes there was a significant association between NICU admissions, neonatal jaundice, and breech presentation with insulin-treated mothers (p=0.06, p=0.003, p=0.004, respectively CI=95%). Among maternal outcomes, there was a significant association between pregnancy-induced hypertension (PIH) and insulin-treated patients (p=0.02 CI=95%), premature rupture of membranes (PROM), and metformin-treated patients (p=0.01 CI=95%) whereas eclampsia was significantly associated with mothers not having GDM (p=0.001 CI=95%).

Conclusion

Based on this preliminary data and considering feto-maternal outcomes, metformin appears to be a safer drug as compared to insulin in the treatment of GDM with more compliance.

## Introduction

Gestational diabetes mellitus (GDM) is defined as diabetes diagnosed during pregnancy that is not clearly overt diabetes. It does not include women with type 1 or 2 diabetes [1]. In other words, it is any degree of glucose intolerance with onset or first recognition during pregnancy [2]. According to small hospital-based studies, 3.2% for GDM and 1.9% for impaired glucose tolerance (IGT) have been reported [3]. The International Diabetes Federation in the year 2019 stated that 16% of live births had some form of hyperglycemia in pregnancy and an estimated 84% were due to gestational diabetes with one in every six births were affected by gestational diabetes mostly common in middle and low-income countries [4]. 

Physiologically, during the last trimester, approximately 7% of women have high levels of glucose in their blood to which their body cannot produce enough insulin to combat [5]. Such pregnancies are considered to be in a diabetogenic state which is a result of the progressive rise in the levels of estrogen, progesterone, human placental lactogen, cortisol, and prolactin with the advancement of pregnancy which is all insulin antagonists [6]. Various factors lead to the development of GDM, e.g., obesity with a BMI above 30kg/m2, high blood sugar, bad cholesterol, hypertension, smoking, lack of exercise, personal history of GDM, and family history of diabetes [1-6]. GDM is associated with both maternal and fetal complications, maternal being pregnancy-induced hypertension, pre-eclampsia, elective cesarean section, polyhydramnios, and fetal outcomes being macrosomia, birth trauma, hypoglycemia, NICU admissions, low birth weight, hypocalcemia and respiratory distress syndrome, and polycythemia [7]. 

Insulin is the gold standard for the treatment of hyperglycemia during pregnancy. However, recent studies have suggested that certain metformin such as glyburide could be safe and be acceptable alternatives. There are no serious safety concerns, and benefits include reductions in neonatal hypoglycemia, maternal hypoglycemia and weight gain, and improved treatment satisfaction [8]. Approximately half of women with a history of GDM go on to develop type 2 diabetes within five to ten years after delivery and are six times more at risk as compared to women who have normal glucose tolerance in pregnancy. Insulin is the recommended first line of treatment if glycemic targets are exceeded, although there is increasing evidence that oral agents (metformin or glyburide) are safe in this situation [9]. However, costs, the number of injections, and feto-maternal outcomes of insulin and metformin necessitate a study to be conducted in this part of the world to determine the glycemic control and compare the feto-maternal outcomes in metformin, insulin-treated GDM patients.

## Materials and methods

Materials and methods

The study setting was at Rehman Medical Institute (RMI), Peshawar with the population comprising women diagnosed with GDM admitted to the obstetrics and gynecologist ward. The inclusion criteria were mothers who were (1) diagnosed with GDM during their pregnancy, (2) diagnosed with GDM during their pregnancy and consented, and (3) mothers who were prescribed insulin, metformin, or collective therapy at RMI during their pregnancy. The exclusion criteria included mothers who were diagnosed with GDM during this pregnancy but did not consent to be part of the study, mothers who had established diabetes mellitus before pregnancy, mothers who had type 1 diabetes mellitus diagnosed during childhood, mothers with the tendency of experiencing high blood sugar levels in stress states and who have become diabetogenic before as well also the mothers at non-prescribed insulin or metformin therapy. The sample size was 150 and the cluster-sampling method was used. The study design was a retrospective study with a duration of January-April 2020.

Data analysis (statistical methods)

Data were retrieved from the Rehman Medical Institute Information and Communication Technology Department (RMI-ICT) which were then entered in SPSS version 21.0 (IBM SPSS Statistics for Windows, IBM Corp., Armonk, NY) where it was analyzed on the basis of variables: demographic data, glycemic control via fasting blood sugar (FBS), oral glucose tolerance test (OGTT), and feto-maternal outcomes. The data was then run through various tests including chi-square, cross tabs, and descriptive statistics. Significant relations with a p-value less than 0.05 were identified which were then presented in the form of tables and graphs along with non-significant results. 

## Results

The sample was composed of patients with no GDM and those who had GDM and were on the following treatment regimens: metformin, insulin, and combined treatment of metformin with insulin (Table 1). Most of them belonged to the 30-40 years of age group, of these, the majority were non-GDM mothers (64) followed by those on metformin (35). Regarding the period of gestation (weeks), the 35-40 week category had the most patients who did not have GDM (64); this category also had the most patients on metformin treatment (35). Similarly, the 20-30 week category had the most patients with no GDM (11) followed by those on insulin treatment (9). Positive family history for GDM was most commonly seen in patients with no GDM (68) followed by those on insulin treatment (21), whereas personal history was most commonly positive for patients on insulin treatment (18), followed by those on metformin treatment (7). Among modes of delivery; C-section was most common in patients who did not have GDM (51), followed by those on metformin treatment (36). Most of the mothers had one child (57) among whom the predominant group was non-GDM mothers (27) followed by those on metformin treatment (17).

**Table 1:** Demographic data of participants

Variables		Treatment			Non-GDM
		Both	Insulin	Metformin	
Age groups	20-30	2	2	8	13
	30-40	5	19	35	64
	40+	0	0	1	1
POAG groups	20-30 weeks	2	9	3	11
	30-40 weeks	5	19	35	64
	40+ weeks	0	0	1	1
History of GDM	Family history	7	21	12	68
	Personal history	5	18	7	12
Mode of delivery	NVD	0	1	4	13
	C-section	6	20	36	51
	Assisted delivery	1	2	6	10
Number of children	1	5	8	17	27
	2	0	1	5	13
	3	0	3	8	14
	4	1	4	4	9

**Figure 1:**
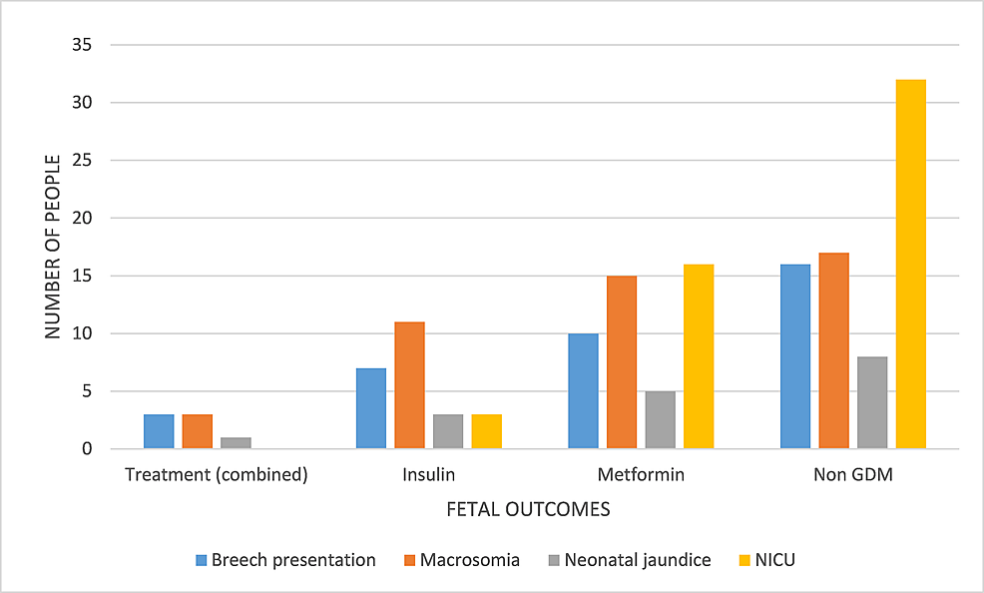
Fetal outcomes

Among fetal outcomes, the predominant outcome was NICU admission (34%), being the most common in insulin-treated mothers (62.7%) followed by metformin-treated (31.3%). Breech presentation (24%) was again most common in insulin-treated mothers (44.4%) followed by metformin-treated (27.7%). Neonatal jaundice (11.3%) was seen mostly in insulin-treated mothers (47.0%) followed by metformin-treated (29.4%). However, macrosomia (30.6%) was most common in metformin-treated mothers (39.1%) followed by non-GDM mothers (30.4%) (Figure 1).

Among maternal outcomes, the predominant outcome was PIH (18%), being the most common among insulin-treated mothers (40.7%) followed by metformin-treated (25.9%). Premature rupture of membranes (PROM) (15.3%) was again most common in metformin-treated mothers (45.8%) followed by insulin-treated (29.1%). PV leak (16%) was also predominant in metformin-treated mothers (41.6%) followed by non-GDM (33.3%). Polyhydramnios (20.6%) was observed equally dominant in insulin and metformin-treated mothers (32.2%). However, eclampsia (13.3%) was most common in non-GDM mothers (50.0%) followed by metformin-treated mothers (25.0%) (Table 2). 

**Table 2:** Maternal outcomes

Maternal outcomes	Treatment			Non-GDM
	Both insulin and metformin	insulin	metformin	
Eclampsia	4	7	12	18
Per vaginal leak	3	4	11	18
Pre-eclampsia	4	9	14	19
PROM	2	13	33	49
Polyhydramnios	7	18	29	43
PIH	5	9	25	45

After applying Pearson's chi-square test on the data, a strong association was seen between PIH and insulin users (p=0.02 CI=95). Another association between PROM and metformin users was observed (p=0.01 CI95). A third association was seen between eclampsia and non-GDM mothers (p=0.001 CI=95) (Table 3).

**Table 3:** Association of insulin, metformin, non GDM with the maternal outcomes

Maternal outcomes	Patients	P values
PIH	Insulin	0.02
PROM	Metformin	0.01
Eclampsia	Non-GDM	0.001

After applying Pearson's chi-square test on the data, a strong association was seen between NICU and insulin users (p=0.03; CI=95). Another association between neonatal jaundice and insulin users was observed (p=0.003; CI=95). A third association was seen between breech presentation and Insulin mothers (p=0.004; CI=95) (Table 4).

**Table 4:** Association of insulin, metformin, non-GDM with fetal outcomes NICU: neonatal intensive care unit

Fetal outcomes	Patients	P-values
NICU	Insulin	0.03
Neonatal jaundice	Insulin	0.003
Breech presentation	Insulin	0.004
Macrosomia	Metformin	0.01

## Discussion

Gestational diabetes mellitus is a growing health issue in many parts of the world. The prevalence of GDM in Pakistani women is comparable to the West but complication rates were higher, possibly due to poorer glycaemic control. Gestational diabetes has serious, long-term consequences for both baby and mother [10]. This study retrospectively evaluated 150 non-GDM to GDM pregnancies on metformin and insulin therapies respectively during pregnancy. The study revealed an increased risk for fetal outcomes on patients with insulin treatment; a significant association was observed between NICU admissions, neonatal jaundice, and breech presentation with insulin-treated mothers. Whereas variable associations were observed in maternal outcomes, PIH and insulin-treated patients, PROM and metformin-treated patients, eclampsia, and mothers not having GDM had significant associations, leading to the overall conclusion that metformin is a safer alternative for the treatment of GDM than insulin.

A cohort study conducted in Karachi in 2010-12 compared the fetomaternal outcomes in patients diagnosed with GDM receiving insulin therapy or metformin and concluded that metformin yielded a better impact on feto-maternal outcomes and glycemic control in comparison to Insulin therapy provided with both less maternal increase in weight and NICU admissions [11]. A meta-analysis conducted in China stated that metformin had statistically significant positive effects on maternal and neonatal outcomes. Metformin may have potential benefits for the mother and fetus [12]. A similar randomized clinical trial conducted in New Zealand and Australia also concluded metformin (alone or with supplemental insulin) to be associated with less perinatal or postnatal complications as compared to insulin. After getting this treatment on a random basis the women even preferred metformin over insulin treatment for later pregnancies [13]. A larger randomized controlled trial that compared metformin with insulin in the treatment of gestational diabetes (MiG trial) suggested that metformin, alone or with supplemental insulin, is an effective and safe treatment option in regards to glycemic control and feto-maternal outcomes [14]. A similar study was conducted at Royal North Shore Hospital, Sydney, Australia on Metformin increasingly being used as a therapeutic option for the management of GDM. They concluded that metformin, by all means, is a safer alternative to insulin [15]. The latest NICE guidelines state that metformin should be offered to women with gestational diabetes if blood glucose targets are not met using changes in diet and exercise within one to two weeks, however, if metformin is contraindicated or unacceptable to the woman only then the women having gestational diabetes be offered insulin [16].

In conclusion, our findings suggest that metformin, alone or with supplemental insulin, is an effective and safe treatment option for women with gestational diabetes mellitus who meet the usual criteria for starting insulin and that metformin is more acceptable to women with gestational diabetes mellitus than is insulin. Further follow-up data are needed to establish long-term safety.

## Conclusions

Metformin showed better effects on feto-maternal outcomes in patients with gestational diabetes in comparison to insulin. Metformin is used through a physiological route, has no storage problem, and nor does it require any dependency. Moreover, it is cheap, easily available, and does not require any needle pricks. With all these features metformin is a better option than insulin in our population for patients having gestational diabetes mellitus. However multiple large-scale studies must be conducted in the future to know better about the two drugs and their feto-maternal outcomes in gestational diabetes.
